# Two Sprayer CVD Synthesis of Nitrogen-doped Carbon Sponge-type Nanomaterials

**DOI:** 10.1038/s41598-018-20079-9

**Published:** 2018-02-14

**Authors:** Emilio Muñoz-Sandoval, Juan L. Fajardo-Díaz, Roque Sánchez-Salas, Alejandro J. Cortés-López, Florentino López-Urías

**Affiliations:** Advanced Materials Division, IPICYT, Camino a la presa San José 2055, Lomas 4a sección, San Luis Potosí, 78216 Mexico

## Abstract

Nitrogen-doped carbon sponge-type nanostructures (N-CSTNs) containing coaxial multiwalled carbon nanotubes are synthesized at 1020 °C by using a modified chemical vapor deposition (CVD) arrangement. Here, the CVD reactor is supplied by two flows coming from two independent sprayers (called sprayer A and sprayer B). The nebulized material in each sprayer is transported by two different gases with different flow velocities. The synthesis of carbon N-CSTNs is performed using different precursors: sprayer A contains a solution composed of ethanol, thiophene and ferrocene, whereas sprayer B contains a solution of benzylamine, thiophene and ferrocene. Samples are classified according to the position inside the reactor and characterized by scanning electron microscopy (SEM), transmission electron microscopy (TEM), X-ray diffraction (XRD), X-ray photoelectron spectroscopy (XPS), Raman spectroscopy and thermogravimetric analysis (TGA). Samples collected at the beginning of the reactor contain curly structures with diameters of 10–100 nm. At the end of the reactor, the sample is mainly formed by one type of structure. A spongy-type material is mainly formed in the hottest zone of the tubular furnace. The N-CSTNs are highly hydrophobic with oil sorption properties, which could be used for adsorption of oil spills.

## Introduction

Single wall carbon nanotubes (SWCNTs) have been one of the most important breakthrough contributions to the technology of materials science^1^. Futaba *et al*., fabricated a macroscopic bulk material that they called SWCNT solid^2^. This material is composed of densely packed SWCNTs, but the bulk material also exhibits the fundamental properties of individual SWCNTs. It was proposed that this material could be used as supercapacitor electrodes. The intention of that research was to build a 3D framework while keeping the individual properties of the building blocks. The ability to connect individual carbon materials to build 3D networks for improving their mechanical and elastic properties is a technology with broad possible applications^3,4^. Carbon sponges are one of the most important carbon materials, with exceptional and diverse properties made with carbon nanotubes as the building blocks^5^. These carbon materials have been used in supercapacitors by different groups. For example, Cheng *et al*., fabricated hybrid flexible α-Fe_2_O_3_/carbon nanotube supercapacitors that presented porous hierarchical structures and high specific capacitances^6^. In this case, the carbon sponges were synthesized by the method outlined by Gui *et al*^7^. and decorated with α-Fe_2_O_3_ using chemical routes. This combination improved the capacitance of the spongy material. Furthermore, He *et al*^8^. combined a skeleton of carbon foam and nitrogen-doped carbon nanotubes to build a 3D carbon hybrid. Nitrogen atoms distributed in the carbon nanostructure were considered as promising electrode materials for supercapacitor applications^8^. Carbon sponges could also be used in the fabrication of long-term rechargeable batteries^9^. Their application in Li-ion batteries is another research topic with great scientific and commercial potential^10,11^. Sharifi *et al*. fabricated 3D architectures decorated with hematite nanorods with 3D morphology to be used as negative electrodes for Li-ion batteries. They also proposed the importance of nitrogen atoms as preferential adsorption sites for the Li^+^ ions^9^. Ke *et al*. introduced carbon nanotubes into Co_3_O_4_ nanowire arrays to build a 3D structure with supercapacitor potentialities. These CNT-oxide nanohybrids showed low conductivity with an enhanced redox reaction, which makes them useful as a high-performance electrode material for energy storage^12^. In addition, materials with sponge morphologies have been proposed for use as shielding material in the GHz range^13^. Using Ni-Cu alloy as a catalyst Ge *et al*^14^. fabricated carbon nanofiber sponges to be used for oil absorption. The sponges were fabricated at 580 °C by CVD using C_2_H_4_ as the carbon source and were tested for oil absorption, showing absorption capacities of up to 75 g/g. The hydrophobic and oleophilic properties of carbon sponges fabricated using the method outlined in Gui *et al*^7,15^. have been used for oil recovery, showing oil adsorption of over 95 wt% after several cycles. Stolz *et al*., used pyrolysis to fabricate a carbon sponge containing a 3D interconnected network with excellent absorption capacities^16^. Using a melamine sponge as a template, Qiu *et al*., fabricated a potential sorbent for the removal of oil from water that also displayed high porosity and hydrophobicity^17^. Another interesting property is their high surface area, which could serve to capture undesirable metals or chemical species in the environment^18^. Wang *et al*., proved that commercial carbon nanotube sponges could be used for solid-phase extraction of organic pollutants at trace levels. They have reported the successful use of these sponges as an adsorbent for the enrichment and analysis of polychlorinated biphenyls at trace levels in water samples^19^. Santangelo *et al*., investigated tensional states due to the interconnections in carbon nanotube sponges fabricated on a Co-Mo-Mg trimetallic catalyst by the decomposition of methane at 900 °C. They concluded that the observed Raman spectra modifications were due to the CNT interconnections^20^. Zhao *et al*., studied boron-doped and nitrogen-doped isotropic 3D CNT frameworks that contained elbow-like junctions with hyperelastic properties. The boron-doped carbon sponges were fabricated with ferrocene, toluene and triethylborane at 860 °C using an aerosol-assisted catalytic CVD method, and the nitrogen-doped materials were made using ferrocene, thiophene and pyridine through a syringe pump procedure^21^. The mechanical properties of carbon sponges are also interesting due to their ability to return to their original form after deformation, which could be important in the fabrication of artificial muscles^22^. Dai *et al*., revealed the highly outstanding mechanical and elastic properties of sponges. The randomly interconnected individual carbon nanotubes grown by CVD that presented super-elasticity, a high strength-to-weight ratio, fatigue resistance, thermo-mechanical stability and electro-mechanical stability^23^. These three-dimensional networks could also serve as scaffolding or a template for growing biological systems such as cells and artificial bone^24^. Using a chemical vapor deposition technique with few precursors and a three-zone furnace, Erbay and collaborators fabricated inexpensive carbon sponges with potential applications in microbial electrochemical cell systems^25^. Wang *et al*., fabricated a sponge-like carbon matrix containing nitrogen and iron to be used in the oxygen reduction reaction as a catalyst. Their procedure involved several steps to synthesize the sponge. However, their ORR catalytic activity apparently exceeded that of Pt/C, probably due to their high pyridinic-type nitrogen content^26^. Thermal annealing was applied to carbon sponges to bring about Fe_2_O_3_ nanoparticles attached to the sponge’s CNTs. They demonstrated that the new structure could be used for applications in catalysis and energy fields^27^. An interesting spongy carbon was fabricated by Barborini *et al*. This spongy carbon was a three-dimensional, highly porous structure that consisted of fully connected sp^2^ negatively curved networks^28^. These three-dimensional networks were synthesized using the chemical vapor deposition (CVD) method in a one-step synthesis strategy^13,29–32^, a hybrid CVD method on templates^33–36^ or through electron beam irradiation at high temperatures^37,38^. Synthesis of carbon sponges was performed by Xue *et al*., where they used copper wires as a substrate and dichlorobenzene and ferrocene as precursors for the CNT sponge growth through CVD^39^. Other routes have been attempted to create macroscopic materials by interconnecting carbon nanomaterials. New technology using spongy carbon materials has also been proposed by several important research groups for unconventional applications. One of the most important goals of nanotechnology is the use of new macroscopic materials constructed piece by piece of nanomaterials, which should show outstanding properties. Carbon nanotube sponges are one member of this category of nanomaterials. For such reasons, the synthesis of carbon nanomaterials must be carefully performed. For example, Liu *et al*., demonstrated that using the same precursor can produce different and complex structures^40^. There is no doubt that among the most important carbon materials nowadays are the 3D carbon networks, including the so-called “sponges”, which have interesting and exceptional mechanical, elastic, catalytic, energetic, and capacitive properties. An enormous effort has been invested to fabricate these types of nanomaterials. Generally, well-aligned multiwalled carbon nanotubes (MWCNTs) are fabricated by CVD using ferrocene (C_5_H_5_)_2_Fe and toluene (C_7_H_8_)^41^. However changes to the electronic structure and the morphology can be obtained by doping. For example, nitrogen in the graphitic layers of carbon nanotubes produces a bamboo structure^42^. A boron atom occupying the place of a carbon atom induces knees and corrugated walls^43^, phosphorous-doping can produce tubes with low oxidation temperatures^44^, oxygen can be trapped on the surface of carbon nanotubes by a functional group to cause solubility^45^, and sulfur can be used to create a Y junction^37,46–54^. Boron-doped carbon sponges also have been successfully fabricated^54,55^. The participation of sulfur in the synthesis of carbon sponges could increase their diameter^50,51,53^. Most of the time, the temperatures for the fabrication of this 3D carbon structures are relatively low (850–1000 °C). It could be important to fabricate these nanostructures at higher temperatures to avoid possible negative effects if they were to be used in high temperature environments. Recently, a carbon sponge-like nanostructure has been prepared at a relatively high temperature (1020 °C) by AACVD using benzylamine as a nitrogen and carbon precursor and ferrocene as the catalyst source^56^. Sulfur was used to promote the building of 3D-architectures. In the present work, nitrogen-doped carbon sponge-type nanostructures (N-CSTNs) are synthesized by a single-step aerosol-assisted chemical vapor deposition method (AACVD) at 1020 °C. The N-CSTNs are fabricated by combining two independent sprayers that contain two different solutions whose respective clouds are carried by different gases and flows. The importance of the present investigation lies in the fact that the concurrence of both the flow and the sources involved in the synthesis can be controlled, resulting in a novel material with important characteristics, as can be seen in the following sections. We demonstrate that the use of the two-sprayer strategy can produce complex or hybrid morphologies with potential application in the electrochemical sensors field^57^, as hybrid reinforcements^58^, or for use with environmental issues^3^, among others. A combination of strategies to fabricate 2D or 3D carbon nanomaterials has been a successful route to producing novel structures with astonishing mechanical, chemical and physical properties^59,60^. Here, we used an ethanol substance with a high oxygen inclusion in the synthesis that helps the benzylamine solution, which acts as a carbon and nitrogen precursor, interact with the iron coming from the ferrocene and thiophene for incorporation of sulfur.

## Methodology

**Figure 1:**
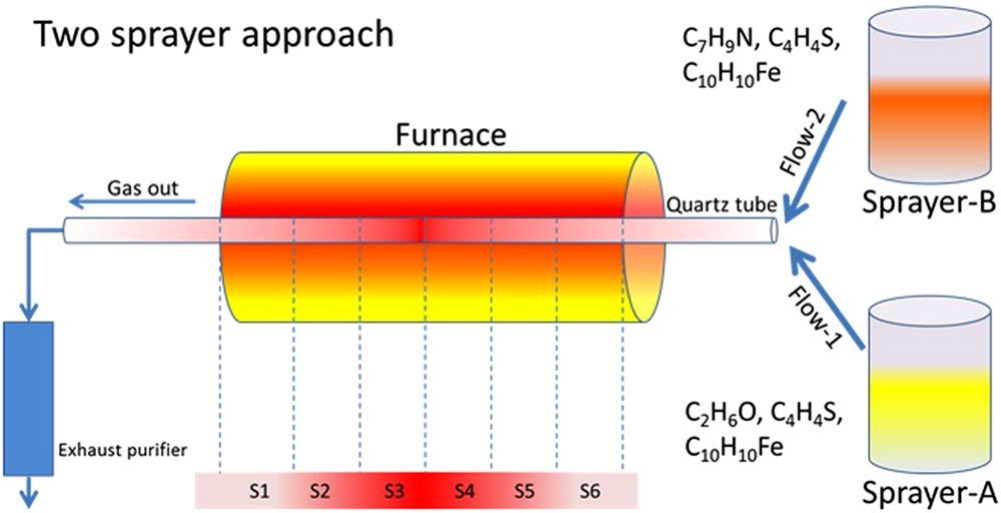
Schematic representation of the chemical vapor deposition setup used to synthesize N-CSTNs. Samples are synthesized at 1020 °C. The resulting material was collected from the quartz tube (reactor) by scraping its inner walls and classifying the sample according to the region in the tubular furnace. The different regions are labeled as S1, S2, S3, S4, S5, and S6. Sprayer (**A**) contains an ethanol solution, whereas sprayer (**B**) contains a benzylamine solution. Both sprayers contain thiophene and ferrocene. More details on the precursor concentrations are given in the text.

N-CSTNs were synthesized using the AACVD technique. Figure 1 shows the approach, which involves pyrolysis using two different sprayers (RBI instrumentation): i) sprayer A, with an ethanol (C_2_H_5_OH) solution containing thiophene (C_4_H_4_S, 0.124 wt%) and ferrocene (Fe(C_5_H_5_)_2_, 1.252 wt%); and ii) sprayer B, with a benzylamine (C_6_H_5_CH_2_NH_2_) solution containing thiophene (C_4_H_4_S, 0.5 wt%) and ferrocene (Fe(C_5_H_5_)_2_, 2.5 wt%). The AACVD experiments were carried out at 930, 960, 990, and 1020 °C for 40 minutes under an inert atmosphere. In the case of sprayer A, following the work of Campos-Delgado *et al*.^61^, Ar gas with a flow of 0.8 L/min transported the cloud. In the case of sprayer B, the gas carrier was a mixture of Ar-H_2_ (95%-5%) with a flow of 1.0 L/min, similar to the case in Ref.^21^. The temperature was controlled by a type 21100 Barnstead/Thermolyne series tubular furnace with one central thermocouple outside the ceramic fiber on the heating chamber to monitor the temperature changes. Inside of this furnace, a quartz tube 1.10 m in length and with an outer diameter of 2.63 cm and 1.5 mm thickness was placed to perform the synthesis. In order to see the modification of the morphology and efficiency, two more experiments were performed changing the flow of sprayer B (0.6 L/min and 1.4 L/min). The two sprayers were connected to the quartz tube by a 24/40 shape glass connecting adapter. The sprayers were turned on at the same time, each forming a cloud of micro-drops after a few seconds, by a piezoelectric piece coupled to an internal hermetic 1-L glass container and an electronic generator (brand RBI ref. 7901). The synthesis of the N-CSTNs began when both clouds entered the furnace. After the catalytic dissolution was pyrolyzed inside the reactor, the organic by-products were cooled by a Liebig condenser and finally were deposited in a bubble gas trap. Figure SI-1a in the supplementary information provides a description of the sprayer and the main parts that were used in our investigations. The resulting material was collected from the reaction quartz tube by scraping its inner walls, and the samples were classified according to different zones in the tubular furnace. Samples were associated with each of the zones and were named sample 1 (S1), sample 2 (S2), and so on, through sample 6 (S6). Scanning and (high-resolution) transmission electron microscopy characterizations were used for morphological studies of the samples. A Helios Nanolab 600 Dual Beam was used to perform the SEM measurements, and the TEM images were taken by an FEI Tecnai F30. A Renishaw micro Raman spectrometer with a 532 nm excitation wavelength was used to evaluate the crystalline structure. The carbon, nitrogen and oxygen contents in the carbon N-CSTNs were measured by XPS (PHI 5000 VersaProbeII). Thermogravimetric analysis was performed using STA 6000 Perkin-Elmer equipment in a temperature range of 50–950 °C with a heating speed of 10 °C/min under dynamic flow of oxygen (20 mL/min). The temperature profile (see Fig. SI-1b) of the furnace was measured cm by cm employing a resistivity scan (RS) pro K type thermocouple stainless steel probe 500 mm in length, 3 mm in diameter, plugged into an RS pro ANSI connector for K type measurements. Steren MUL-600 hardware and software were used to acquire the data on a PC interface (see Fig. SI-1c).

## Results and Discussion

**Figure 2:**
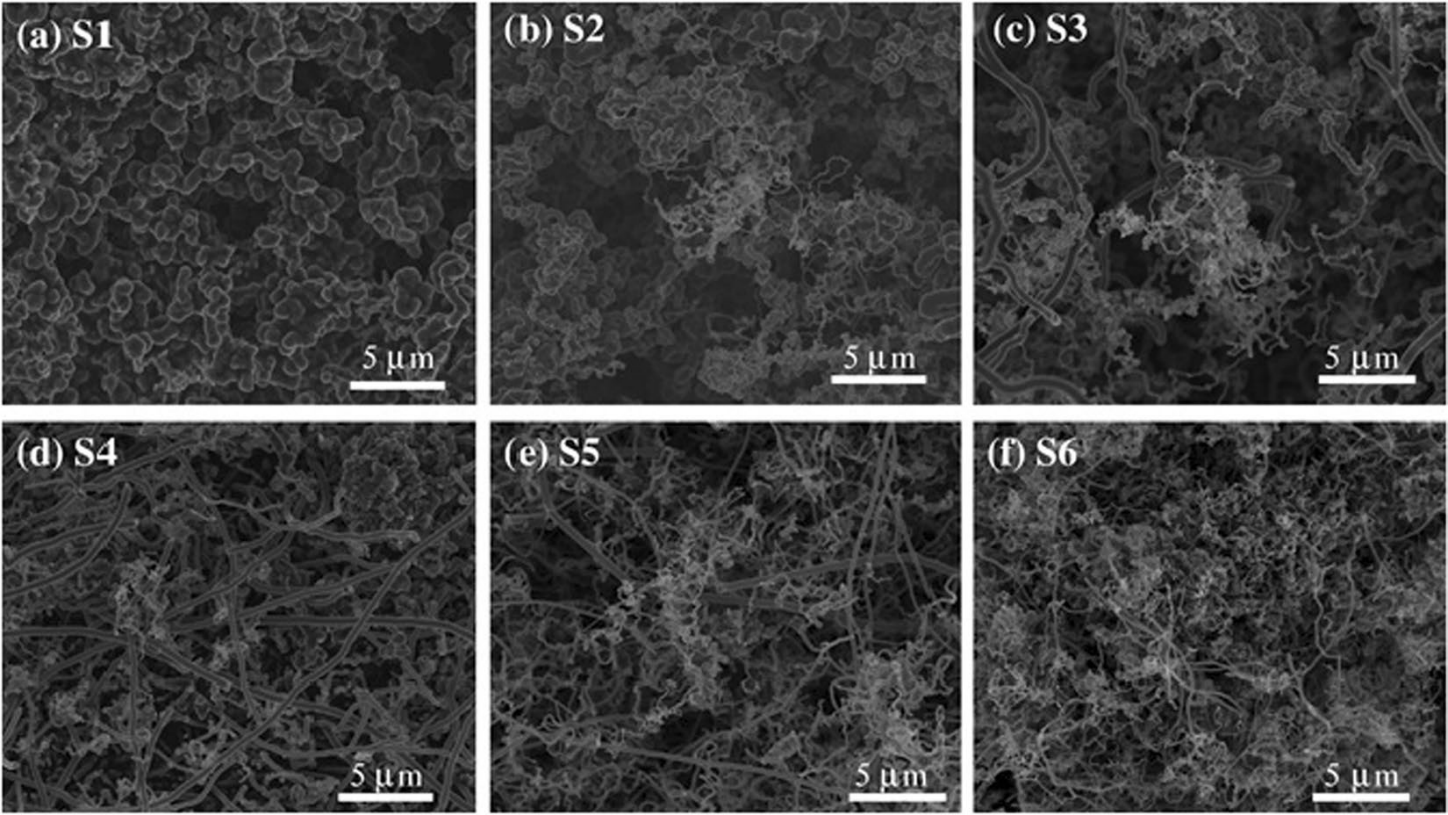
SEM images of samples produced at 1020 °C with 40 min of growth. The images correspond to the different furnace zones: S1, S2, S3, S4, S5, and S6 (see Fig. 1). Note that depending on the furnace zone, the collected material exhibits different morphologies and sizes in the carbon structures. The hottest zones correspond to zones 3 and 4. High magnification images can be seen in Fig. SI-1.

Figure 2 depicts SEM images of samples synthesized at 1020 °C during a 40-min growth period. High-magnification SEM images are also shown in the supplementary Information (Fig. SI-2). Different morphologies can be observed depending on their position alongside of the reactor. The robustness of the carbon structure increases as we move away from the reactor inlet, as shown by the difference between sample S6 (reactor inlet) and sample S1 (reactor outlet) in Fig. 2. Furthermore, unlike in the single-sprayer approach (previous work reported in Ref.^56^), the two-sprayer setup promotes the growth of diverse types of carbon structures and morphologies in each reactor zone. This diversity of structures is richer in morphologies for samples close to the reactor inlet. For example, in sample S1 (Fig. 2a), just at the end of the tubular furnace, the morphology of the N-CSTNs is formed by aggregates or chains that are extremely curved and apparently constructed from individual enormous spherical nanoparticles made of graphite material with large diameters of approximately 600 nm, also shown in Fig. SI-2a. In sample S2 (Fig. 2b), graphite spherical nanoparticle aggregates are present in smaller quantities and zigzagged-corrugated carbon nanotubes with diameters of approximately 50 nm appear, as shown in Fig. SI-2b. For the hottest zone (the center part of the tubular furnace), seen in sample S3 (Fig. 2c), the amount of coalesced graphite spherical nanoparticles almost disappears, and zigzagged-corrugated carbon nanotubes and robust long carbon fibers (LCFs) with diameters of approximately 400 nm emerge, as shown in Fig. SI-2c. The largest amount of spongy material was found in samples rich in LCFs. Therefore, we believe that LCFs are the most important component of the N-CSTNs. Figure 2d shows the different morphologies found in sample S4. The LCFs are more frequently observed, but with slightly reduced diameters. These LCFs are accompanied by carbon tubular structures with small diameters and zigzagged-corrugated carbon nanotubes that are distributed throughout the entirety of sample S4 (Fig. SI-2d). In sample S5, there are also LCFs with small and large diameters and graphite nanoribbons (Fig. 1e). The graphite nanoribbons can be seen in Fig. SI-2e. In addition to the morphologies found in sample S5, sample S6 contains wavy carbon nanotubes (Fig. 1f and Fig. SI-2f). Most of the N-CSTN samples are concentrated in sample S3 (46.1 mg), S4 (58.8 mg) and sample S5 (87.8 mg). In general, we found a strong dependence of the carbon nanostructure morphologies with the growth zone, and the sample with the highest yield of LCFs material was sample S4. If the gas flow of sprayer B (benzylamine solution) is deceased, the yield of sample S2 is considerably increased, due to the more abundant material. In contrast, if the gas flow is increased to 1.4 L/min, the sample with the highest weight is sample S5. The morphology of each of the samples is similar for the three different flow cases. Figure SI-3 shows the photos of each sample and their respective weight profiles. We also found that the synthesis temperature plays an important role. Figures SI–(5–9) show the SEM images of samples produced at 930 °C, 960 °C, and 990 °C. It is clear that with a reduction in the synthesis temperature, other carbon nanostructures can emerge.

**Figure 3:**
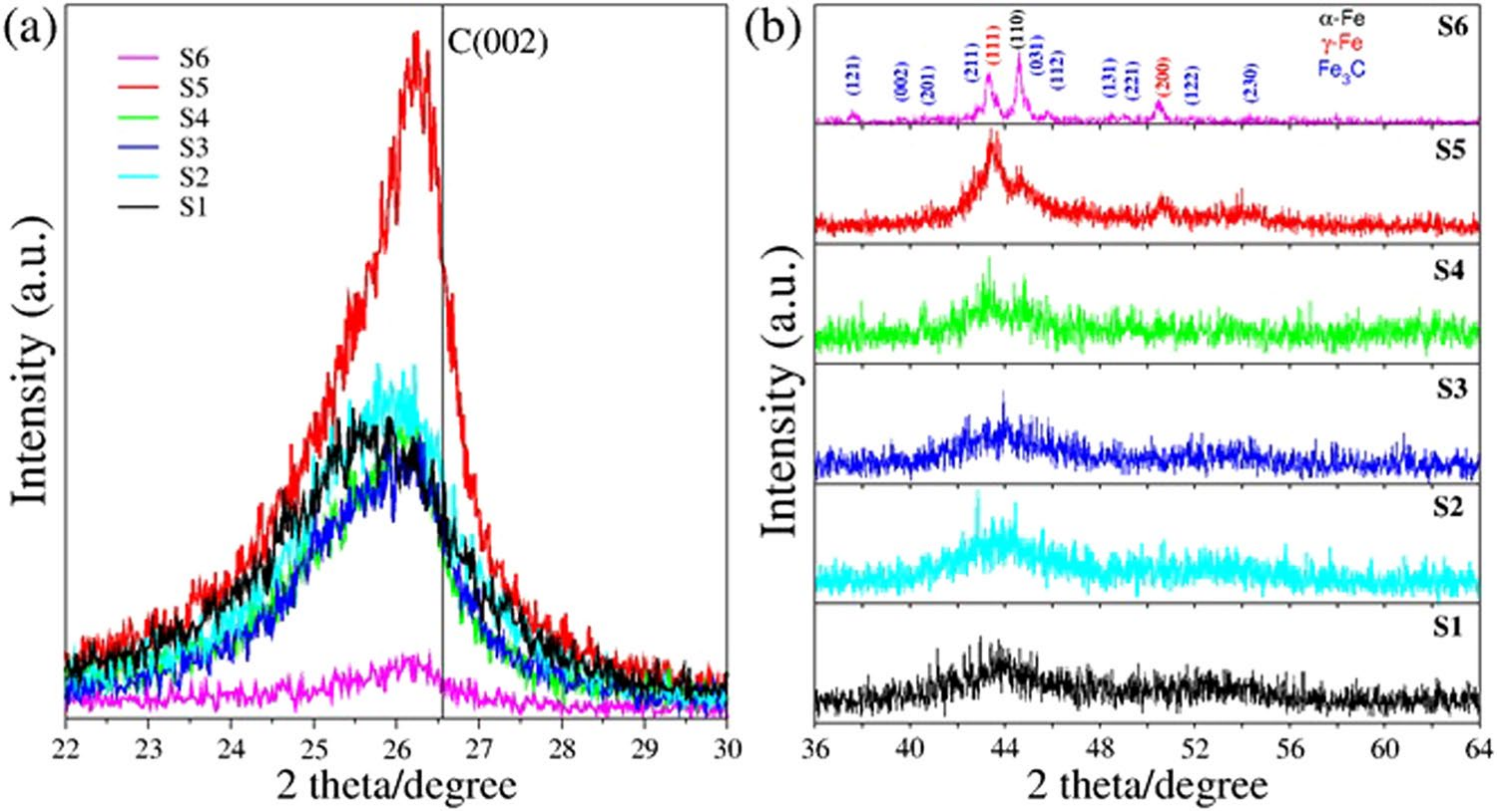
XRD patterns of samples collected from inside the furnace, grown at 1020 °C for 40 min. (**a**) The C(002) graphitic peak, designated by a vertical line, corresponds to highly oriented graphite. The asymmetric shape of this peak suggests the presence of at least two types of graphite materials. A deconvolution analysis of the C(002) plane can be seen in Table SI-1 and explained in the text. (**b**) XRD signals attributed to Fe_3_C, α-Fe, γ-Fe, and graphite.

Figure 3 depicts the XRD characterization results for N-CSTNs. Figure 3a shows the (002) graphite peak for all samples. The peak features, such as intensity, angle position, and broadness, are different for all samples. The peaks are shifted to lower angles than the ones they appear at with graphite. Furthermore, the peaks exhibit an asymmetric shape, suggesting the presence of at least two different graphite layer materials. To elucidate this point, we performed a deconvolution analysis using two curves (Gaussian and Lorentz) to study the different types of layered materials involved in the (002) graphite peak. The lower angle peak is called gamma (γ), whereas the higher angle peak is called pi (π), an example of which can be seen in Fig. SI-4 for sample S4. In general, the interlayer distances associated with the gravity center of the γ-peak yields ~3.5 Å, whereas the π-peak yields ~3.4 Å. Note that the interlayer distance in graphite is 3.34 Å. Most graphite materials with an interlayer distance of ~3.4 Å could be attributed to very well-ordered graphite layers, whereas the interlayer distance of ~3.5 Å could be attributed to defected graphite or turbostratic graphite layers. The integrated area under the Gaussian curves provides an estimation of the amount of the two involved different materials corresponding to the γ-peak (turbostratic graphite or defected graphite layers) and the π-peak (ordered graphite layers). An interesting competition between disordered and ordered graphite layers can be observed for the different samples (see Table SI-1 for details). Note that in sample S4 where LCFs are abundant, the deconvolution yields that 83% of the sample corresponds to disordered graphite layers. The catalytic materials in the N-CSTNs were determined to be Fe_3_C, α-Fe, and γ-Fe phases (Fig. 3b). Sample S6 exhibits a well-defined XRD pattern perfectly showing these phases. Despite the fact that the XRD patterns for samples S1-S4 are noisy, it is also possible to distinguish the peaks representing the main planes of the Fe_3_C, α-Fe, and γ-Fe phases. This fact could mean that the catalytic nanoparticles reduced their size.

**Table 1:** Deconvolution analysis data for the C1s spectrum.

Sample	C1s-XPS	Gravity center	FWHM	Integrated area %
S1	Fe-carbide sp^2^-carbon sp^3^-carbon C-OO-C=O	283.61 284.56 285.38 286.40 288.75	0.51 0.97 1.02 1.51 3.95	2.5 68.8 17.7 7.4 3.9
S2	Fe-carbide sp^2^-carbon sp^3^-carbon C-O O-C=O	282.75 284.03 285.01 285.69 286.97	0.5 1.23 0.72 1.15 2.95	0.5 80.4 8.3 7.2 3.6
S3	Fe-carbide sp^2^-carbon sp^3^-carbon C-O O-C=O	283.41 284.17 284.92 285.68 288.56	0.77 0.87 0.88 1.71 5.09	6.1 58.2 13.2 12.1 10.4
S4	Fe-carbide sp^2^-carbon sp^3^-carbon C-O O-C=O	283.96 284.62 285.28 286.48 288.45	1.04 0.85 1.20 1.40 3.46	9.4 48.9 29.9 8.3 3.5
S5	Fe-carbide sp^2^-carbon sp^3^-carbon C-O O-C=O	283.58 284.40 285.32 286.40 288.29	0.78 0.91 0.97 0.97 4.23	5.0 66.9 16.8 4.5 6.8
S6	Fe-carbide sp^2^-carbon sp^3^-carbon C-O O-C=O	283.12 284.12 285.12 286.05 288.42	0.40 1.03 0.87 1.25 4.22	1.7 69.5 13.2 7.5 8.1

Results for samples S1, S2, S3, S4, S5, and S6. The sp^3^ signal could be attributed to the presence of organic ether (R-O-R) and organic ester (RCOO-R) groups attached to the surface of N-CSTNs.

**Figure 4:**
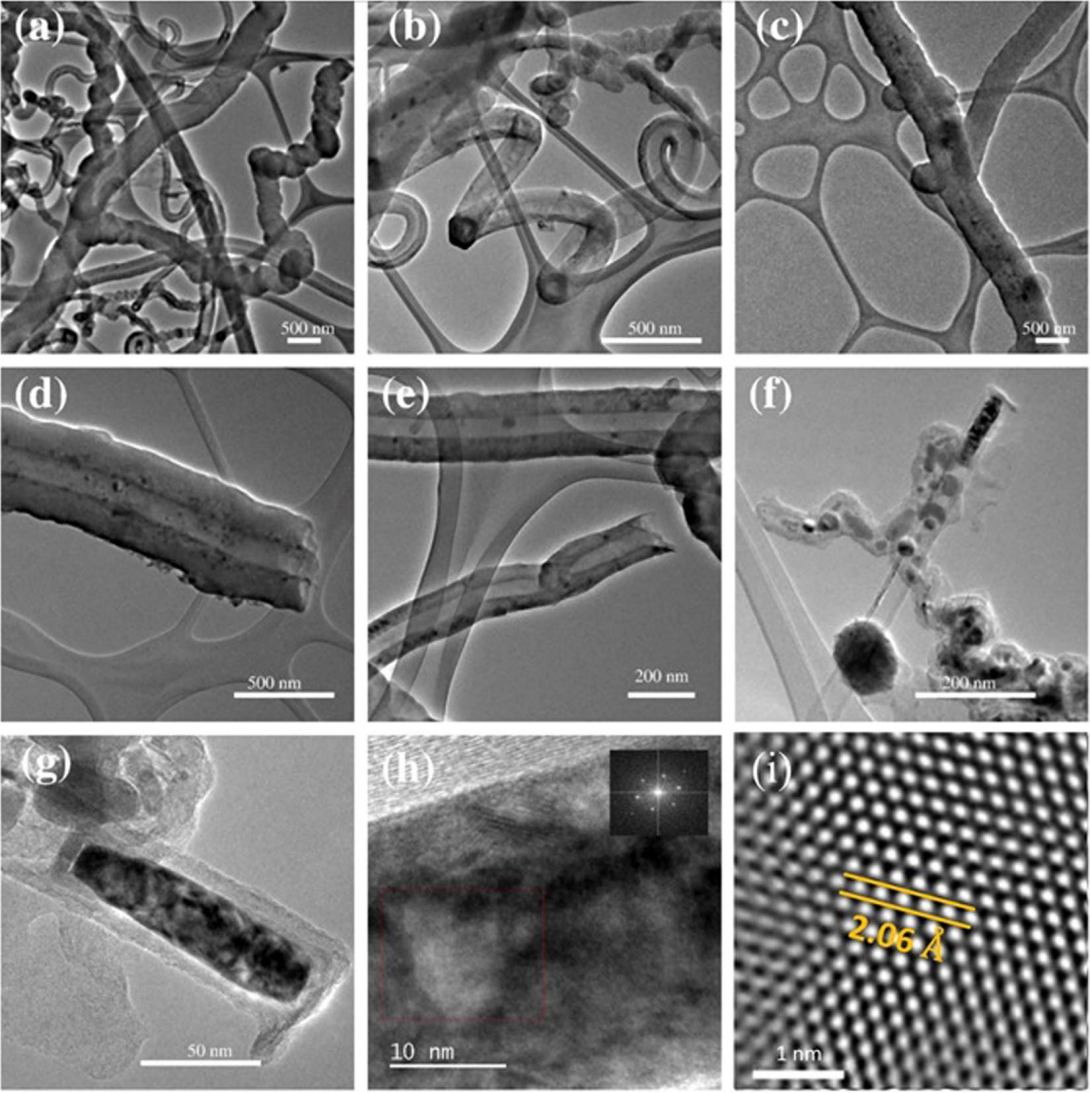
TEM images of a sample synthesized at 1020 °C for 40 min. The sample was obtained from the S4 zone. (**a**) Junctions formed from carbon nanotubes. (**b**) Graphitic nanoribbons with rolled-up edges. (**c**) Carbon nanotubes with anchored metal nanoparticles. (**d**), (**e**), and (**f**) close-up images of carbon nanotubes. (**g**) and (**h**) HRTEM images of metal nanoparticles inside carbon nanotubes. (**i**) FFT refinement from the enclosed area shown in (**h**). The determined interlayer distance corresponds to 2.06 Å, which is attributed to the crystallographic plane (−1 1 1) of α-Fe.

**Figure 5:**
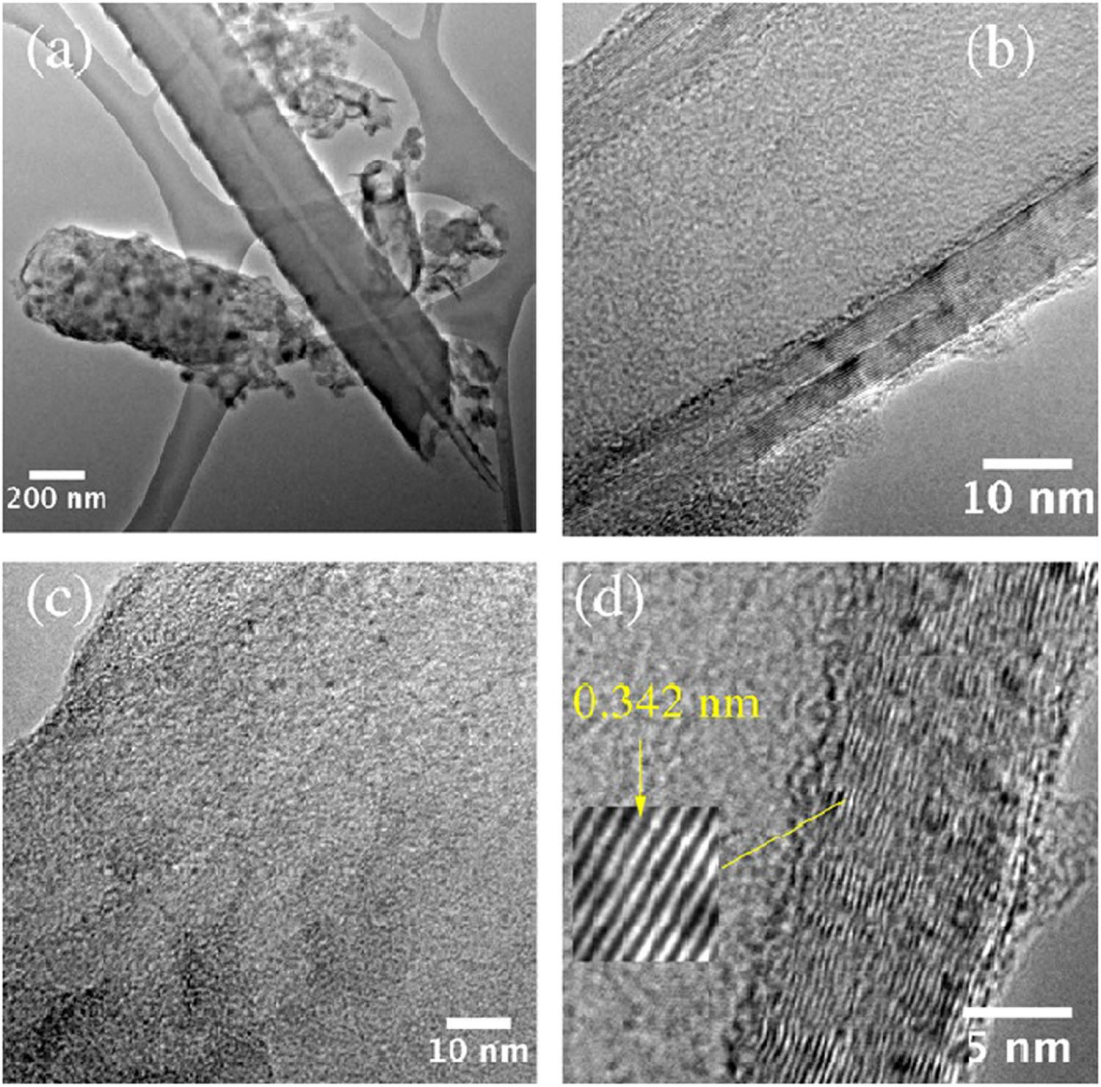
HRTEM images of the samples synthesized at 1020 °C and grown for 40 min. The sample was obtained from zone S4. (**a**) Typical samples found in the N-CSTN materials, which consist of N-MWCNTs with a high graphitization surrounded by graphite layers with a poor graphitization (coaxial-like carbon fibers). (**b**) Image showing the innermost layers. (**c**) Image showing the outermost layers. (**d**) Image showing the interlayer distance between the carbon layers.

Figure 4 shows TEM images corresponding to sample S4. Fiber sponges, junctions, zigzagged, helical and wavy nanotubes can be observed in Fig. 4a. For example, a magnification of a wave carbon fiber is shown in Fig. 4b, where it is possible to observe the larger internal diameter and internal bamboo-shaped compartments. Due to the large diameter, it is possible that these carbon fibers collapsed to form graphite nanoribbons. A large diameter carbon fiber with attached large metal nanoparticles surrounded by graphite materials is depicted in Fig. 4c. These nanoparticles could act as a catalyst to start the growth of new carbon fibers, thus producing the branches observed in N-CSTNs. Figure 4(d–e) shows a very small material anchored to its surface and several black spots distributed alongside the nanotube. The internal tubular hole of these types of nanotubes does not present a bamboo shape. Figure 4f illustrates three types of carbon structures: i) the structure of a zigzagged nanotube with several metal nanoparticles inside, ii) a straight few-layer carbon nanotube with a large metal nanoparticle at the tip, and iii) a carbon fiber joined to a metal nanoparticle. Figure 5(g–h) shows a high magnification image of the elongated nanoparticle found at the tip of the carbon nanotube. This nanoparticle is not monocrystalline (Fig. 5h). It was not possible to define unique zone axes for this nanoparticle. The inset in Fig. 5h shows the corresponding fast Fourier transformation (FFT). Through an image refinement process employing the inverse fast Fourier transformation (IFFT), the interlayer distance (Fig. 5i) was determined to be 2.06 Å, which corresponds to the plane (−1 1 1) of α-Fe.

Figure 5 shows TEM and HRTEM images of a carbon fiber found in sample S4. Figure 5a displays the TEM image of the core-shell carbon fiber with its innermost ordered graphite layers surrounded by disordered outermost graphite layers. Another type of carbon structure found in the N-CSTNs is the graphite nanoribbons, which are also present in Fig. 5a (lower left). At this magnification, this carbon material shows several defects, likely due to the nitrogen doping and embedded Fe, Fe_3_C-nanoparticles, or due to small Fe clusters intercalated in the graphite layers. Figure 5b displays well-aligned innermost graphite layers (high degree of graphitization) and a large shell composed of not-well-layered carbon material (Fig. 5c). The diameter of the internal carbon nanotubes is approximately 60 nm and contains around 30 carbon layers (Fig. 5d). Additional HRTEM images showing the disordered outermost graphite layers can be seen in Fig. SI-10.

**Figure 6:**
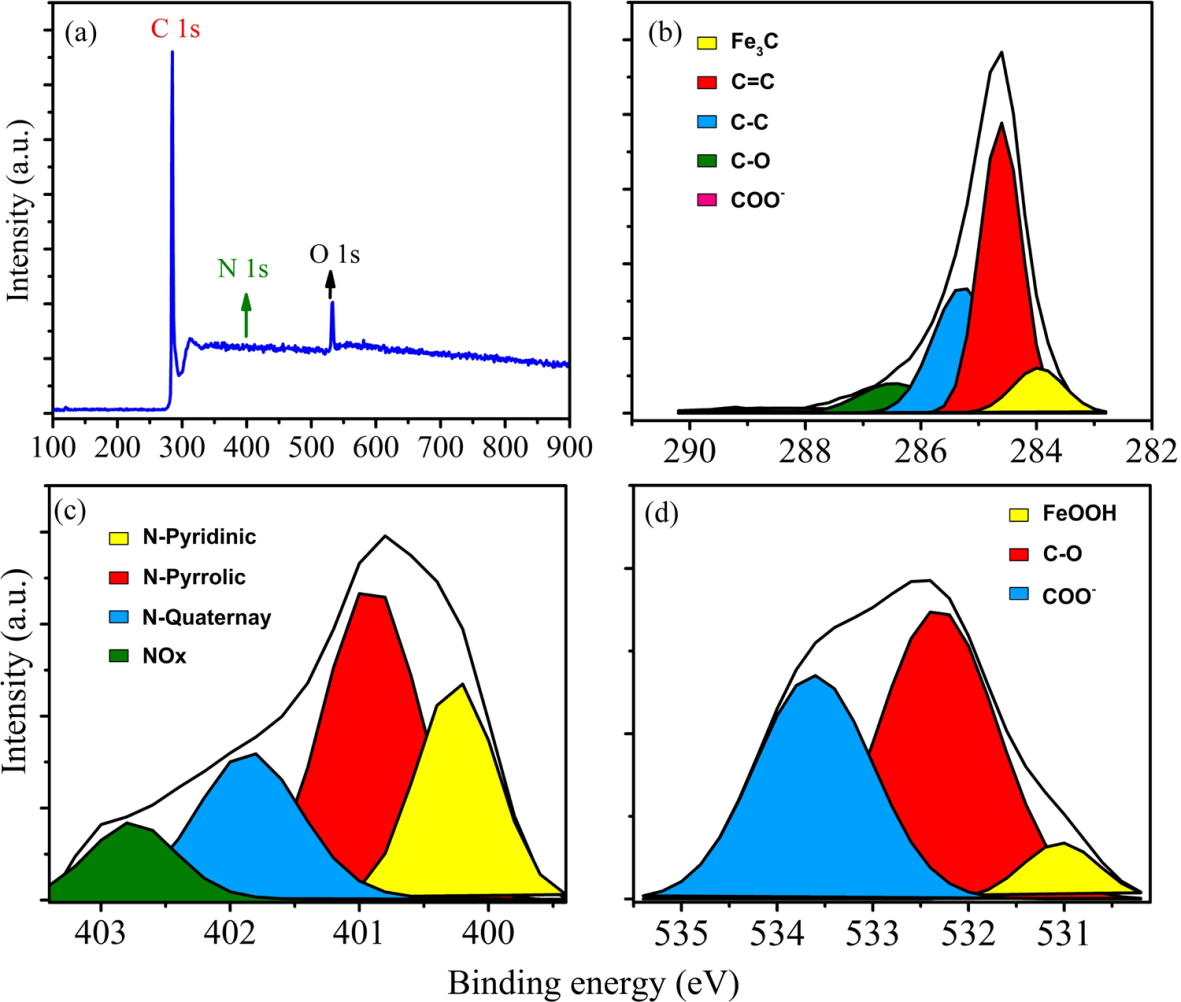
(**a**) Survey XPS spectrum of sample S4 with the peaks related to C1s, N1s and O1s; (**b**) high-resolution C 1s peak, (**c**) high-resolution N 1s peak, and (**d**) high-resolution O1s peak.

Figure 6 exhibits a typical XPS survey spectrum of N-CSTNs and their respective C1s, N1s and O1s in high-resolution spectra along with the deconvolution. The sample in question is S4, which is one of the samples that presents a macroscopic spongy appearance. Deconvolutions were performed considering the chemical species O-C=O 3.5% (288.4 eV), C-O 8.3% (286.4 eV), C-C 29.9% (285.2 eV), C=C 48.9% (284.6 eV) and Fe_3_C, iron carbide 9.4% (283.9 eV) for C1s (Fig. 6b). In the case of N1s (Fig. 6c), the chemical species were N-O nitrogen-oxygen (402 eV), N-quaternary (401 eV), N-pyrrolic (400.3 eV) and N-pyridinic (398.6 eV). High-resolution spectrum of O1s (Fig. 6d) was built using O-C=O 40.7% (533.6 eV), C-O 52.7% (532.3 eV), and Fe_2_O_3_ iron oxide 6.6% (531 eV) chemical bonding. Similar considerations were employed to study the other samples.

**Figure 7:**
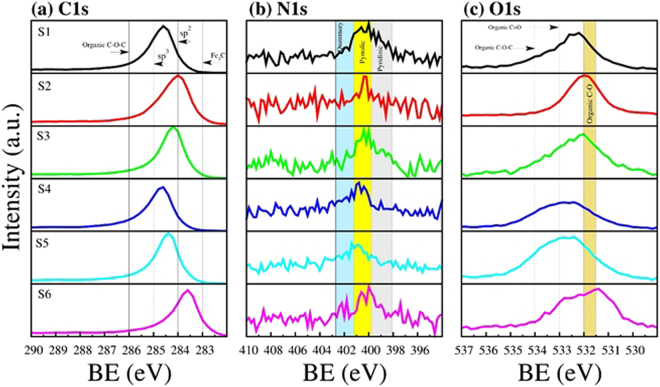
X-ray photoelectron spectroscopy (XPS) analysis results for N-CSTNs. (**a**) The C1s line scan shows a sp^2^-hybridized C signal and a broad shoulder containing a signal coming from oxygenated carbon groups and C−O groups. (**b**) The N1s line scan exhibits a broad N signal, which could be composed by quaternary N (401.2–402.2 eV), pyrrolic N (399.8–401.2 eV), and pyridinic N (398.1–399.8 eV). (**c**) The O1s line scans show the presence of an ether C-O bond.

Figure 7 shows a comparison of the XPS analysis of the six samples. The gravity center and the broadness of the peaks of C1s, N1s, and O1s core-level X-ray photoelectron spectra depend on the sample type. For example, in the C1s spectrum, samples S1, S4, and S5 contain a high amount of sp^3^ carbons (~285 eV), shown in Fig. 7a. In these cases, the samples contain robust structures with large diameters (Fig. 2a,d and e). Samples S1, S2, and S6 seem to exhibit more sp^2^ carbons when compared to graphite material. The deconvolution data can be seen in Tables 1–3 showing the gravity center, full width at half maximum (FWHM) and the integrated area (%). The integrated area provides an estimation of the degree of hybridization and functionalization of the sample^58^. The results revealed that samples S2 and S6 present the highest degrees of sp^2^ hybridization with measurements of approximately 80.4% and 69.5%, respectively (Table 1). Conversely, samples S3 and S4, revealed the lowest sp^2^ concentration with 58.2% and 48.9%, respectively. The deteriorating contribution of the graphite material was due to the increment of the sp^3^ hybridization reaching up to ~29.9% in sample S4. It is worth mentioning that samples S3, S4, and S5 are formed mainly by LCFs, with S4 being the sample that mostly contains LCFs. The deconvolution analysis showed that the carbon atom may be joined to oxygen atoms via single and double bonds (C-O, O-C=O). These are probably part of ether and ester groups (~286 eV), carboxyl groups (~288 eV) and less influenced by carbonyl groups (~287 eV), and these signals of single and double bonds were remarkable in sample S3. The presence of the Fe-C signal from iron-carbide materials was clearly detected for samples S3, S4 and S5, reaching up to 9.4% of the material. This fact was also confirmed by the XRD results. Figure 7b depicts the XPS result for the nitrogen spectra. The main peak was found around the binding energy of 401.5 eV where the nitrogen can be incorporated into the graphite lattice in quaternary, pyrrolic, and pyridinic chemical bonding states. The N1s spectra in all cases demonstrated that nitrogen is mainly incorporated into the sample in a pyrrolic fashion. The results of the deconvolution analysis are listed in Table 2. The highest contribution of pyrrolic nitrogen was found in samples S1, S2 and S4 with percentages of approximately 67.5%, 75.2% and 68.1%, respectively. Regarding samples S5 and S6, the three types of N-doping are competitive. The estimated percentages shown in Table 2 are correlated to the sample morphology. For example, samples S1, S2 and S3 exhibit a great amount of zigzagged carbon fibers, which could contain higher concentrations of pyrrolic nitrogen, whereas sample S4 is formed mainly by robust wavy and straight carbon nanotubes, which could contain higher concentrations of quaternary nitrogen. XPS results for the O1s spectra are shown in Fig. 7c, revealing that the width and position of the main peak depend on the type of sample. Samples S1, S2, S3, and S6 present a shift towards the organic C-O binding energy, whereas samples S4 and S5 exhibit broad peaks shifted towards organic C=O/COO^−^ binding energies. The presence of metal FeOOH (530.5 eV) can be detected in almost all of the samples (samples S1 to S4). The deconvolution analysis data for the O1s peak can be seen in Table 3. Based on the XPS results, the super hydrophobicity of the N-CSTNs could be related to the presence of organic C-O and C=O functionalities associated with ether and ester groups, respectively^60,61^, where a steric impediment is generated as a consequence of the incorporation of these types of functional groups to the defected carbon fiber surface. We found that the hydrophobicity in samples S4 and S5 is due mainly to ester compounds, probably in acetate groups, whereas in samples S1, S2, and S3, it is due to ether groups, probably in an ethoxy radical, and in both cases, oxygen atoms are bonded to the carbon surface.

**Table 2:** Deconvolution analysis data for the N1s spectrum. Results for samples S1, S2, S3, S4, S5, and S6.

Sample	N1s-XPS	Gravity center	FWHM	Integrated area %
S1	Pyrrolic-N Pyridinic-N	400.60 399.04	1.84 1.72	67.5 32.5
S2	Quaternary-N Pyrrolic-N Pyridinic-N	401.19 400.31 399.32	0.60 0.90 0.48	9.5 75.2 15.3
S3	Quaternary-N Pyrrolic-N Pyridinic-N	401.83 400.31 398.47	0.59 1.82 1.57	3.3 66.5 30.1
S4	N-O Quaternary-N Pyrrolic-N	402.75 401.84 400.67	0.91 0.92 1.21	13.3 18.6 68.1
S5	N-O Quaternary-N Pyrrolic-N Pyridinic-N	403.37 402.24 400.96 399.76	0.89 1.32 1.20 0.95	12.1 25.6 48.5 13.8
S6	N-O Quaternary-N Pyrrolic-N Pyridinic-N	402.65 401.20 400.45 399.53	1.40 0.51 0.95 0.60	31.4 16.5 43.3 8.8

The following ranges of binding energy were considered for the peak assignation. Quaternary-N: 401.2–402.2 eV, pyrrolic-N (399.8–401.2 eV), and pyridinic-N: 398.1–399.8 eV. Samples S4, S5, and S6 exhibit a signal around 403 eV, which is attributed to N-O bonds.

**Table 3:** Deconvolution analysis data for the O1s spectrum.

Sample	O1s-XPS	Gravity center	FWHM	Integrated area %
S1	FeOOH C-O C=O	530.69 532.18 533.44	0.76 1.41 1.72	4.4 61.5 34.1
S2	FeOOH C-O C=O	530.79 531.97 532.96	1.93 1.28 1.29	14.2 71.2 14.7
S3	FeOOH C-O C=O	530.64 532.15 533.59	1.28 1.20 1.49	13.3 20.3 66.4
S4	FeOOH C-O C=O	531.04 532.32 533.61	0.94 1.45 1.44	6.6 52.7 40.7
S5	C-O C=O	532.12 533.50	1.66 1.89	59.8 40.2
S6	C-O C=O	532.01 533.48	1.78 1.37	71.3 28.7

Results for samples S1, S2, S3, S4, S5, and S6. The C-O bonds may be attributed to the presence of ethoxy or hydroxyl groups. The presence of the C=O bond signal suggests the presence of ester, carbonyl, or carboxyl groups.

**Figure 8:**
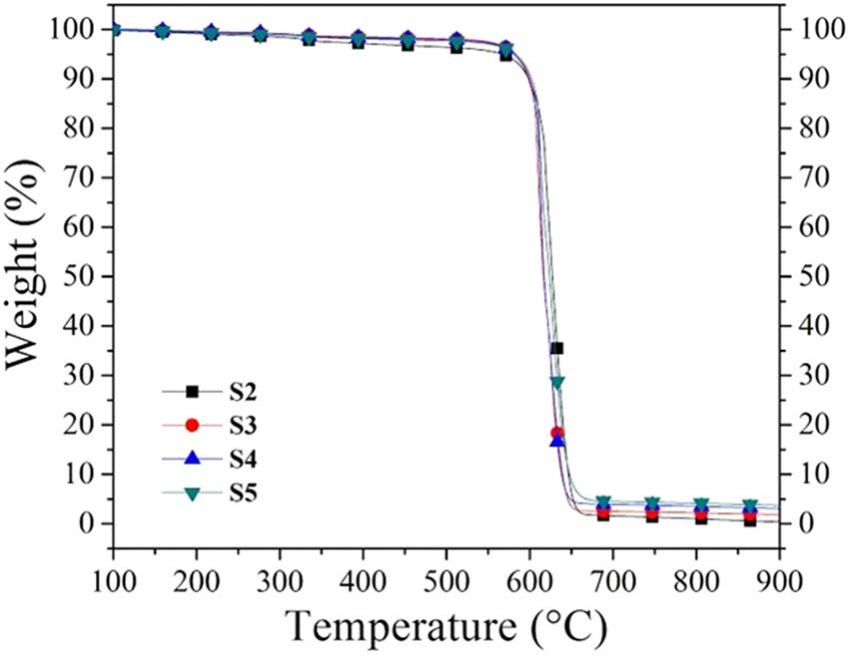
Thermogravimetric analysis of N-CSTNs. The onset temperatures displayed by the N-CSTNs are similar to those found in carbon nanotubes. The residual or remaining material at the high temperature limit is larger for samples collected close to the entrance of the reactor. XRD characterizations have shown that the residual contains different iron oxide materials. This result suggests that the ferrocene used as a precursor in the CVD experiment is mainly decomposed close to the entrance of the reactor.

TGA results for samples S2, S3, S4, and S5 are shown in Fig. 8. The oxidation temperatures are around 620 °C, which is higher than that of typical carbon nanotubes (~520 °C)^44^. In general, at the beginning of the oxidation process, the samples start to lose weight slowly (3–5%) during the first 500 °C. This behavior could be due to the decomposition of functional groups and amorphous carbon oxidation. All analyzed samples are degraded almost entirely in the range of 590 °C to 650 °C, and after this decrement, non-appreciable changes in the TGA curve were observed. The amount of residual material increases proportionally as the sample is collected close to the furnace entrance. For example, in sample S2 (far from the reactor entrance), the residual is ~2%, whereas in sample S5 (close to the reactor entrance), the residual is ~5%. This trend is consistent with the XRD characterization, where narrow peak signals related to Fe- or Fe_3_C-nanoparticles are easily observed in samples collected close to the furnace entrance (samples S4, S5, and S6). This fact indicates the large sizes of the nanoparticles containing Fe.

**Figure 9:**
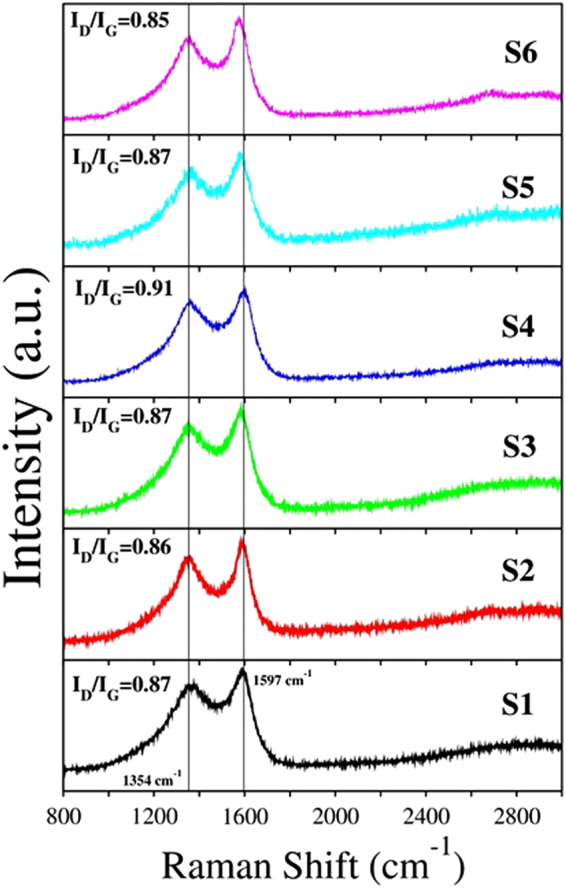
Raman spectra from different synthesized samples using 532 nm (1.958 eV) laser as an excitation source. The D- and G-bands at 1354 cm^1^ and 1597 cm^1^, respectively, for graphite are indicated by the vertical line. The ratio of the intensity of the D- Raman peak to the G- Raman peak (I_D_/I_G_) is indicated for each sample. The non-null value of the I_D_/I_G_ ratio indicates the presence of defects in the graphite layers.

Raman spectroscopy characterizations revealed that N-CSTNs exhibit the typical D and G bands of graphitic materials (Fig. 9). The D-Raman band is related to defects in the graphite materials (edges, vacancies, N-doping), whereas the G-Raman peak is attributed to ordered graphite. The ratio of the intensity of the D-Raman peak to the G-Raman peak (I_D_/I_G_) is indicated for each sample. Values of I_D_/l_G_ < 1 indicate that the N-CSTNs are formed mainly by ordered graphite. The highest value of I_D_/I_G_ was found for sample S4. This fact is concomitant with the XPS results, where the concentration of the carbon sp^3^ hybridization of sample S4 is higher. We have also found that samples S3, S5, and S6 exhibit a downshift in the G-Raman peak. This downshift may be associated with the innermost and outermost layers of the LCFs. Also the emergent downshift could be due to the presence of nitrogen doping in the graphite layer, as confirmed by XPS analysis.

**Figure 10:**
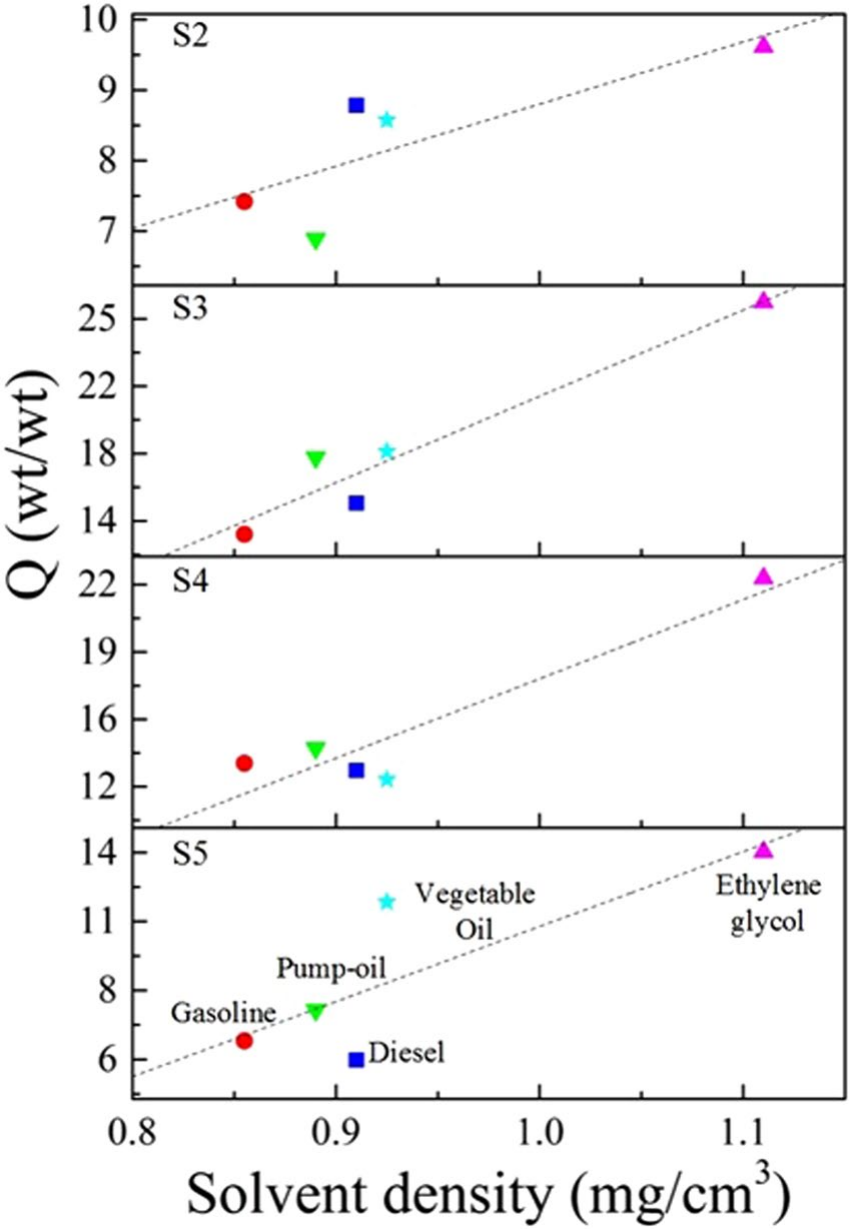
Absorption capacity for different oils/solvents measured from N-CSTNs. (**A**) direct relation between the solvent density and the absorption capacity is observed. Sample S3 yields the maximum absorption capacity above the 25% weight (with ethylene glycol).

All samples exhibited a super hydrophobic behavior similar to other reported carbon sponges^7,31^. However, the origin of the super hydrophobicity in carbon sponges is far from clear. We propose a possible origin of the super hydrophobicity based on our XPS results. The nature of the C-O and C=O bonds is important. It is unlikely that the C-O bonds are associated with phenolic groups (hydrophilic) since the samples are hydrophobic. Then, C-O bonds may be attributed to hydrophobic groups such as epoxy groups or parts of ethoxy groups. On the other hand, C=O bonds could not be attributed to carbonyl or carboxyl groups, since these are hydrophilic. It is likely that C=O bonds are contained in ester groups. The ester and ethoxy groups attached to the N-CSTNs could create a steric impediment where all of the surface charges are homogeneously distributed on the N-CSTNs and promote a superhydrophobic behavior. The hydrophobicity in sample S3 could be mainly due to ester groups, whereas in samples S1, S2, S4, S5, and S6, the hydrophobicity is probably due to the existence of ethoxy groups (Table 3). The absorption capacity of the N-CSTNs was also tested on different oil/solvents, shown in Fig. 10. The test was carried out for gasoline, diesel, pump-oil, vegetable oil, and ethylene-glycol. It was found that sample S3 absorbed 25 times its own weight for the ethylene-glycol and approximately 15 times its own weight for all other considered solvents.

## Discussion

N-CSTNs present different morphologies depending on the zone where they were extracted. It seems that the main morphology is LCFs that are composed of two types of materials, namely, one is a high crystalline core carbon nanotube and the other is composed of thick shell disordered graphite layers, which wrap the crystalline carbon nanotubes. Unlike carbon sponges synthesized with a single sprayer^56^, the spongy materials synthesized using the two-sprayer approach exhibit a less-thick disordered carbon shell with a high graphitization of graphite layers of the core carbon fiber (see samples S3–S5 in Fig. 2). Another significant difference is the appearance of a high percentage of pyrrolic-N in the N-CSTNs. This pyrrolic-N doping could live in the outermost graphite layers of the carbon fibers. An exception is found only in sample S4, where the quaternary-N is more abundant (57.7%). Conversely, in carbon sponges synthesized with a single sprayer, sample S4 contains mainly quaternary-N. Also, in the single-sprayer approach, the pyrrolic-N and pyridinic-N are competitive, which is also the case in sample S2, but in sample S1, the competition is between pyrrolic-N and quaternary-N^56^. In general, the two-sprayer approach promotes long carbon fibers with different diameters, zigzagged carbon fibers, and graphite ribbons. We found that carbon sponges fabricated using the two-sprayer approach are more thermally stable than those synthesized with a single sprayer. The oxidative temperatures derived from the TGA yielded 620 °C and 610–670 °C for the double- and single-sprayer approaches, respectively. The absorption capacity of N-CSTNs considerably decreased with respect to the absorption reported in Ref.^56^. This fact could be associated with the existence of organic ethoxy and ester groups on the N-CSTN surface that avoid the absorption of different oil/solvents.

## Conclusions

We have synthesized CSTNs using a two-sprayer approach in an AACVD method. The CSTNs were classified according to where they grow inside the reactor. We demonstrated that the two-sprayer approach promotes the growth of coaxial carbon fibers, zigzagged carbon fibers, and graphite nanoribbons close to the reactor entrance. XPS revealed that all of these structures are doped with nitrogen. Furthermore, the diameter of the carbon fibers increased for samples synthesized far from the reactor entrance. The best sponges were found in the hottest zone of the reactor (sample S4). These are formed mainly by coaxial long carbon fibers, 300 nm in diameter. A deconvolution analysis of the (002) peak provided two different peaks associated with the 3.4 Å and 3.5 Å interlayer distances. It was also found that the sample formed mostly of coaxial carbon nanotubes contains 83% of the expanded graphite material and has an appreciable amount of pyrrolic nitrogen. The absorption properties of one sample were tested with oil and solvents, reaching an absorption capacity of 25 times its own weight. The N-CSTNs were shown to be super hydrophobic, similar to sponges fabricated with a single sprayer. This study provides important information on carbon sponges classified inside the reactor. For instance, if one requires a carbon sponge rich in N-pyrrolic for a specific application, samples collected from sample S4 are recommended.

## Electronic supplementary material

Supplementary Information
