# Supplementary material for: Two Sprayer CVD Synthesis of Nitrogen-doped Carbon Sponge-type Nanomaterials
Source: Sci Rep. 2018 Feb 14;8:2983. doi: 10.1038/s41598-018-20079-9 (PMC5813181; doi:10.1038/s41598-018-20079-9)
Supplement: Supplementary file 1 — Supplementary Information [file 41598_2018_20079_MOESM1_ESM.pdf]

Two Sprayer CVD Synthesis of Nitrogen-doped Carbon Sponge-type Nanomaterials

Emilio Muñoz-Sandoval, Juan L. Fajardo-Díaz, Roque Sánchez-Salas, Alejandro J. Cortés-López, Florentino López-Urías*

SUPPLEMENTARY INFORMATION

Table SI-1: Emilio Muñoz-Sandoval et al.

Sample	Gravity center	$d_{002(\pi)}$, $d_{002(\gamma)}$	Intensity	FWHM	Integrated area (%)
**S1**	25.3896<br>25.7462	3.5038<br>3.4561	63.1583<br>93.3769	4.1886<br>1.8834	59.7872<br>40.2127
**S2**	25.4242<br>25.8764	3.4991<br>3.4390	58.0761<br>125.7751	4.1934<br>1.7451	52.3053<br>47.6946
**S3**	25.4117<br>26.0002	3.5008<br>3.4229	63.0158<br>85.9908	3.2631<br>1.4833	61.4370<br>38.5629
**S4**	25.6148<br>26.1461	3.4735<br>3.4041	101.2546<br>70.6698	2.4456<br>0.7152	83.0379<br>16.9620
**S5**	25.6232<br>26.2399	3.4724<br>3.3922	176.3092<br>215.8753	2.6763<br>0.8100	72.9321<br>27.0678
**S6**	25.3036<br>26.0876	3.5155<br>3.4116	10.4392<br>17.4289	4.8717<br>1.0324	71.9558<br>28.0442

Table SI-1: Data from the deconvolution (two bands) C(002) peak: γ-band (first-row, red color) and the π band (second row, black color) for the different samples labeled as S1, S2, S3, S4, S5, and S6. The angle (2θ) peak position (gravity center), the interlayer spacing (d_{002}), the peak intensity (Intensity), the full width at half maximum (FWHM) and the area under the curves are all presented. The integrated area of the π band is related to the number of aromatic atoms or graphitization, whereas the integrated area of the γ band indicates the quantity of disorder in the graphitic structures.

Figure SI-1: Emilio Muñoz-Sandoval et al.

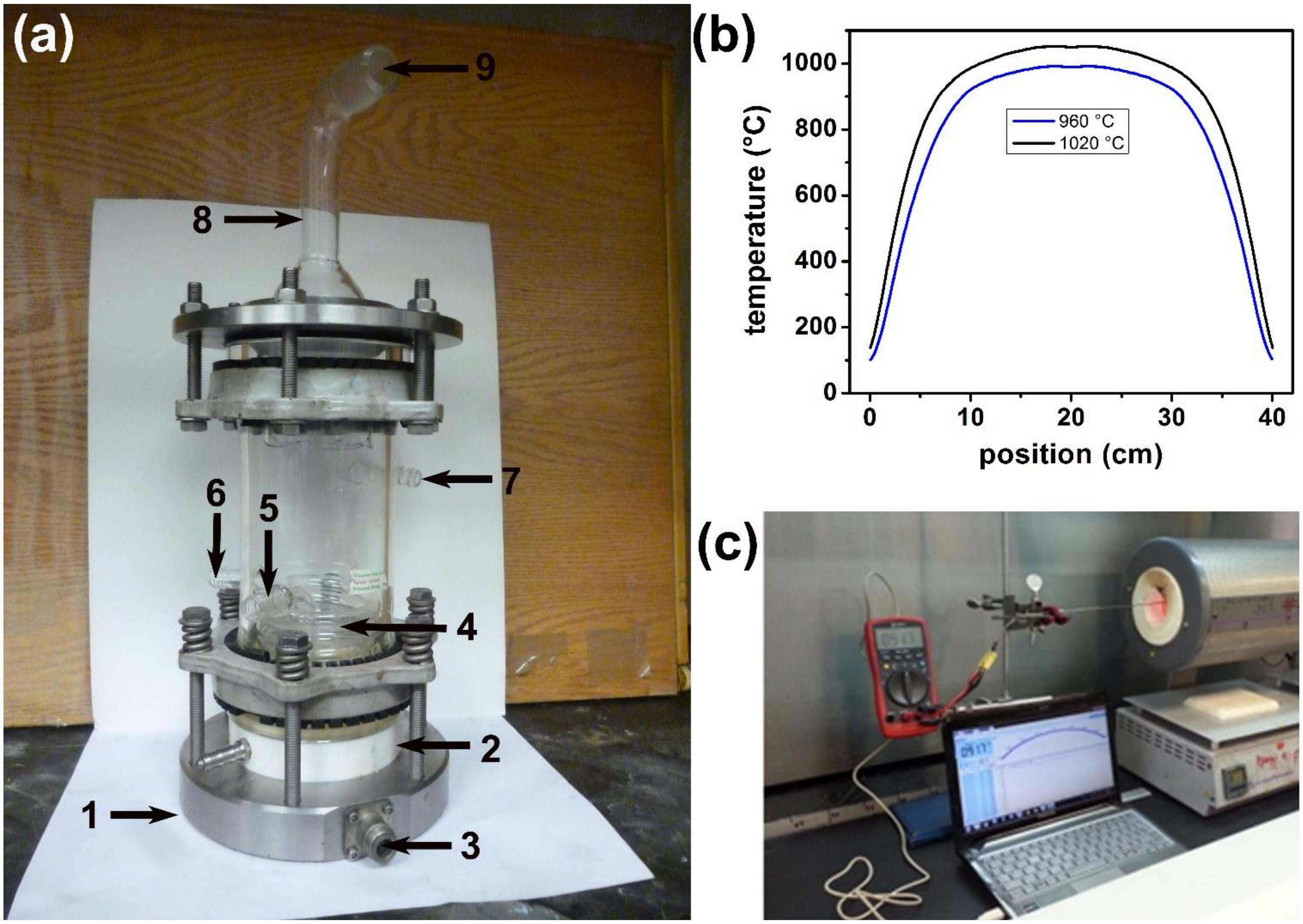

Figure SI-1: **(a)** Typical sprayer used to fabricate carbon sponges or carbon nanostructures: 1) steel base containing the piezoelectric transducer; 2) Teflon base of Pyrex glass container IL (QVF-80/200 20 cm length and 8 cm of ID); 3) plug for the electronic generator connector; 4) spiral water cooler system; 5) and 6) water inlet and water outlet; 7) gas carrying input adapter; 8) a 90°-bent glass adapter; 9) gas outlet to be connected to the quartz tube. **(b)** Temperature profile obtained from the tubular furnace used in the fabrication of N- CSTNs. **(c)** Set up to scan the tubular furnace temperature profile.

Figure SI-2: Emilio Muñoz-Sandoval et al.

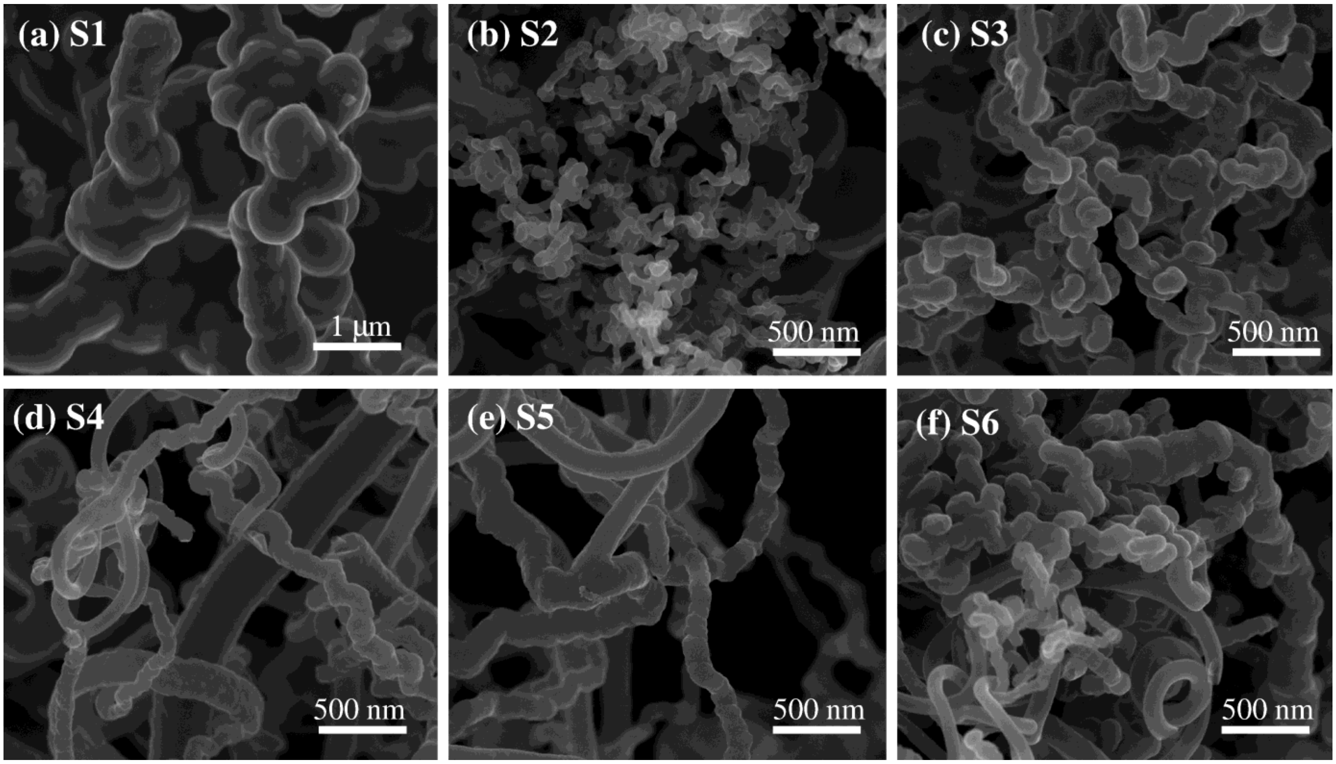

Figure SI-2: SEM images for samples S1-S6 (high magnification). Morphology and size changes of the carbon nanostructure can be identified inside the reactor.

Figure SI-3: Emilio Muñoz-Sandoval et al.

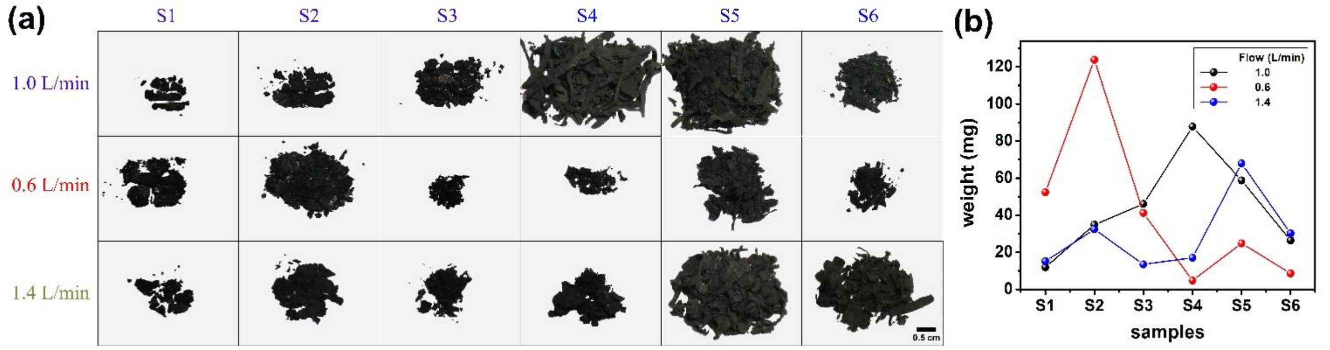

Figure SI-3: Photograph of different carbon materials (S1 to S6) obtained from zones 1 to 6 in distinct conditions of flow **(a)** along with their respective weight profiles **(b)**. In all cases, the spongy material is S5. However, depending on the flow used, the spongy macroscopic aspects are S4 and S5, S5, and S5 and S6 for flows of 1.0, 0.6 and 1.4 L/min, respectively.

Figure SI-4: Emilio Muñoz-Sandoval et al.

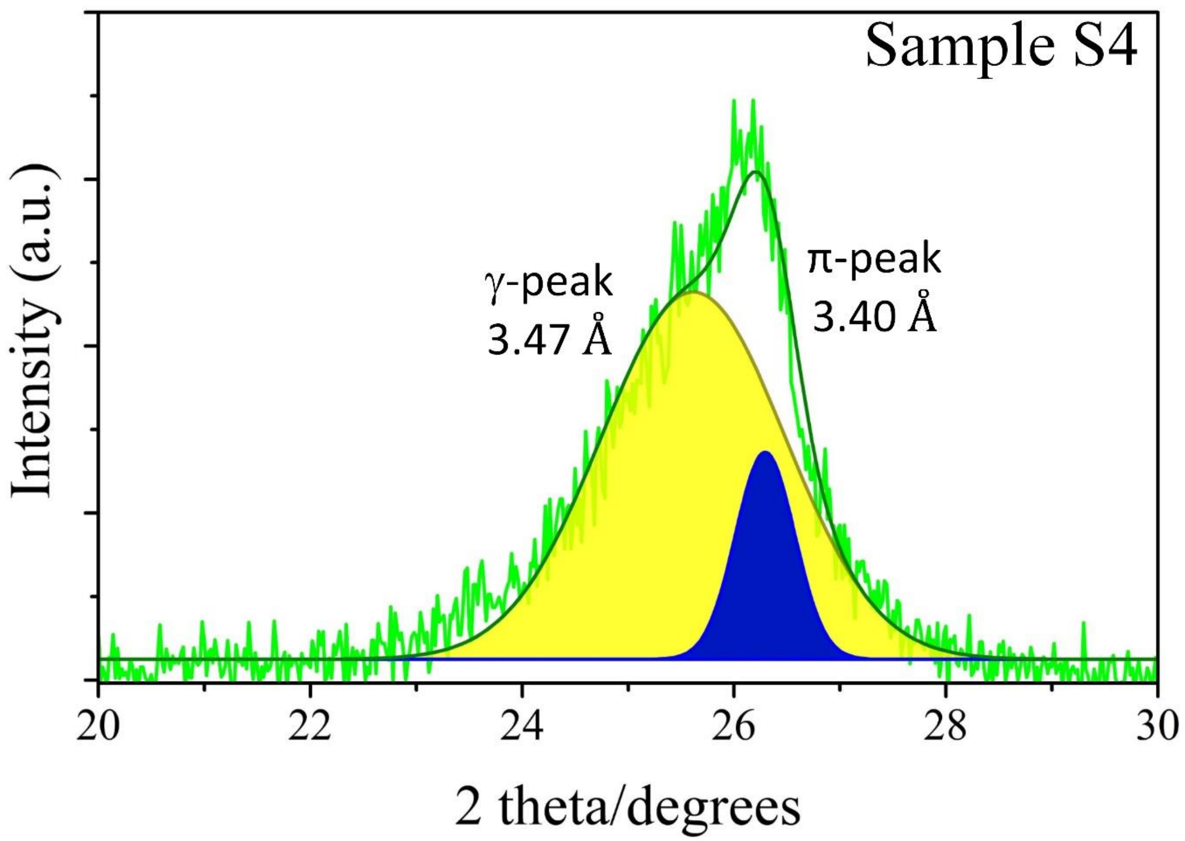

Figure SI-4: Deconvolution analysis of the (002) graphite peak. The lower angle peak is called gamma (γ), whereas the higher angle peak is called pi (π). Gaussian and Lorentz curves are used. The results for sample S4. The interlayer distance associated with each peak is indicated.

Figure SI-5: Emilio Muñoz-Sandoval et al.

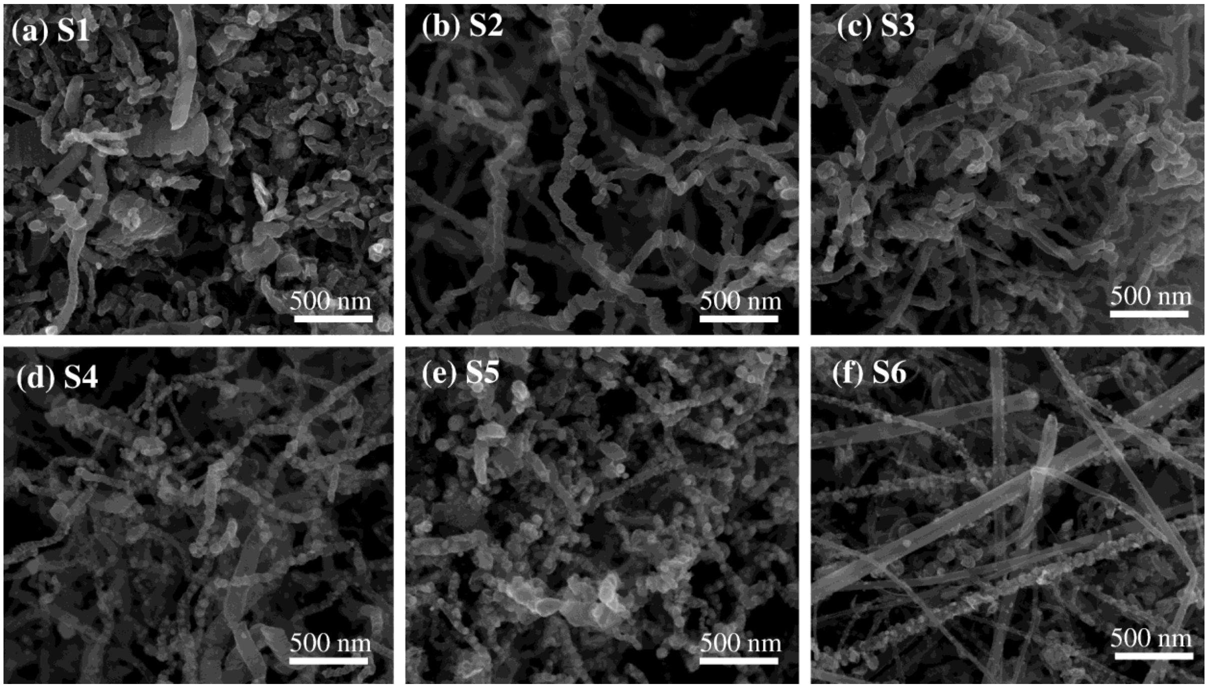

Figure SI-5: SEM images of samples S1-S6 synthesized at 930 °C.

Figure SI-6: Emilio Muñoz-Sandoval et al.

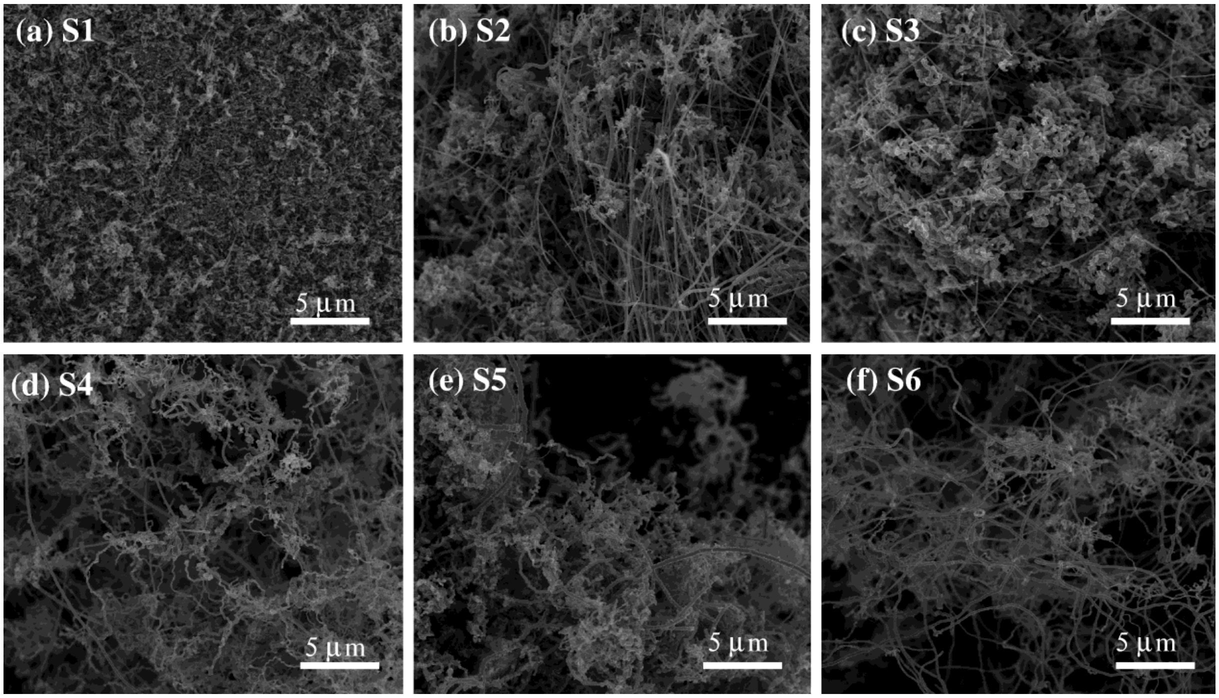

Figure SI-6: SEM images of samples S1-S6 synthesized at 960 °C.

Figure SI-7: Emilio Muñoz-Sandoval et al.

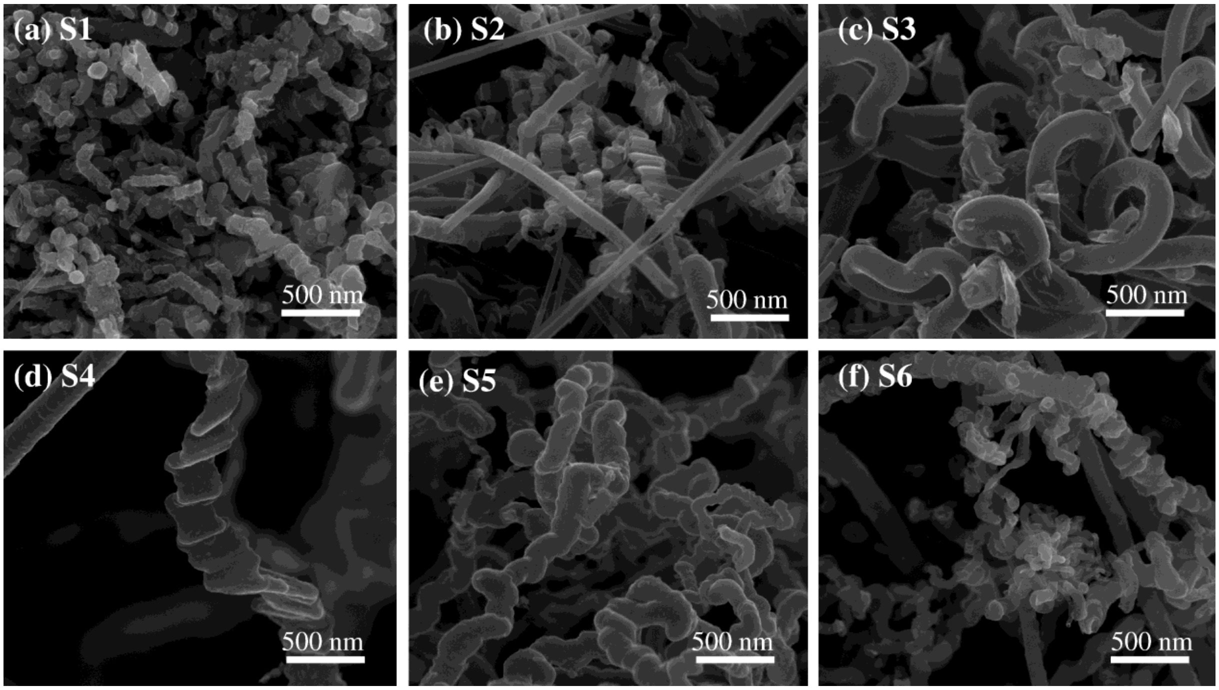

Figure SI-7: SEM images of samples S1-S6. Samples synthesized at 960 °C (high magnification).

Figure SI-8: Emilio Muñoz-Sandoval et al.

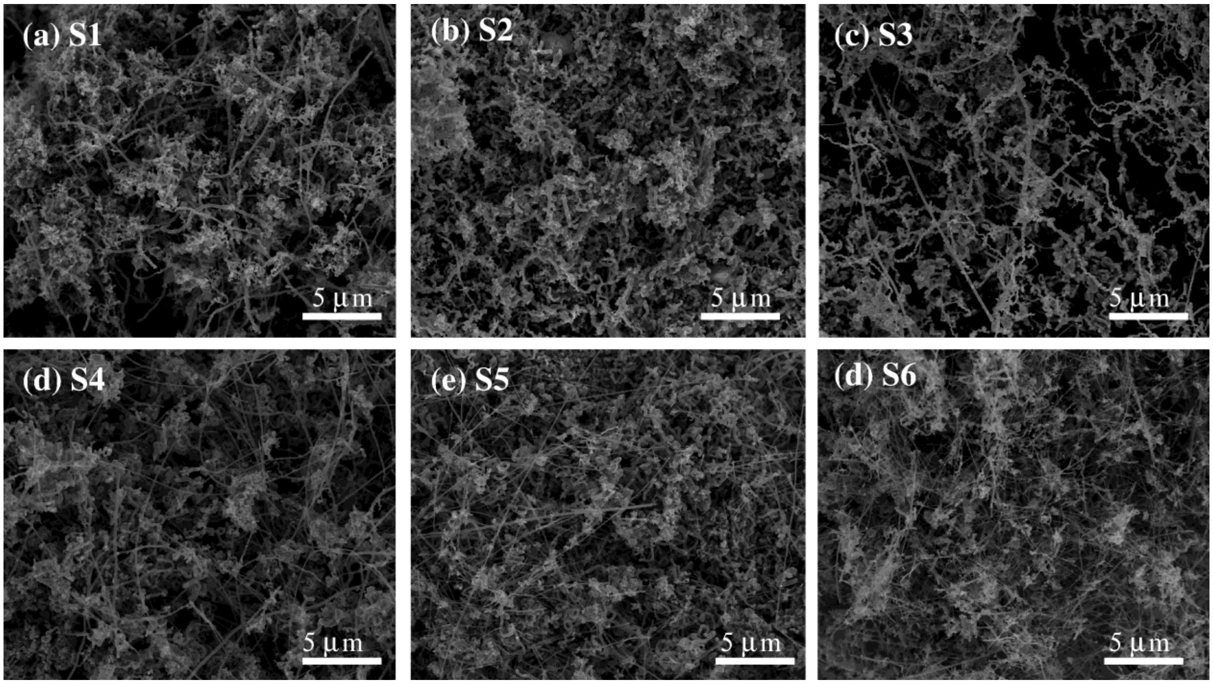

Figure SI-8: SEM images of samples S1-S6 synthesized at 990 °C.

Figure SI-9: Emilio Muñoz-Sandoval et al.

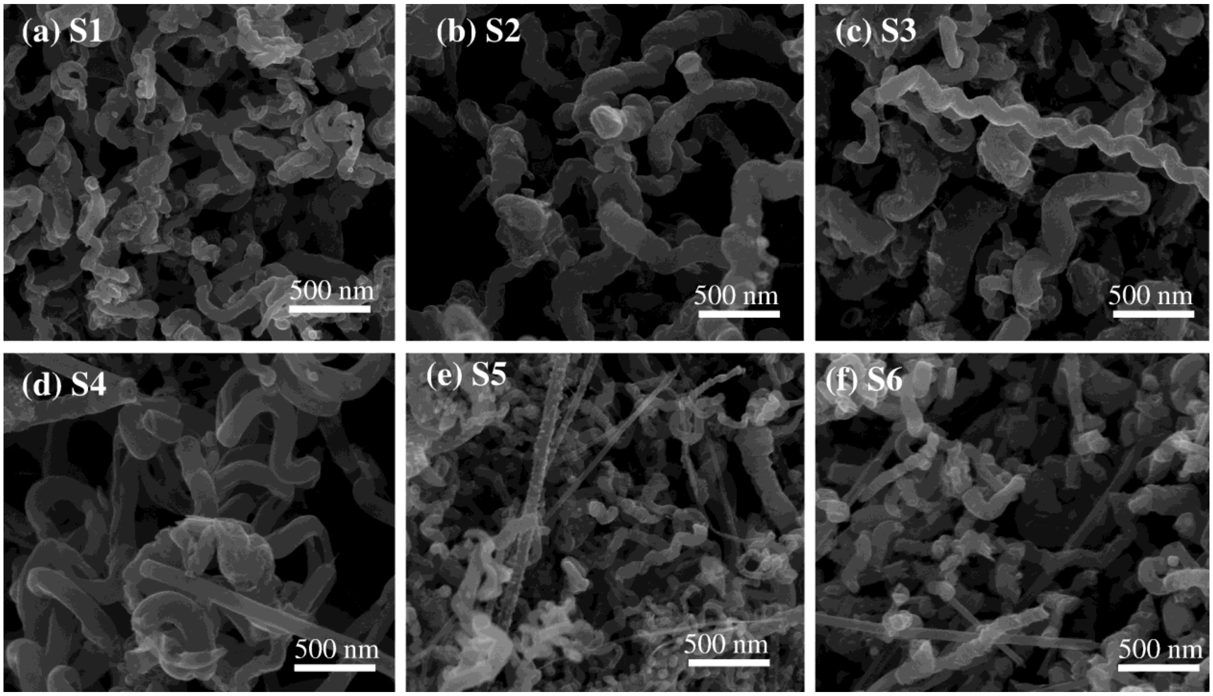

Figure SI-9: SEM images of samples S1-S6 synthesized at 990 °C (high magnification).

Figure SI-10: Emilio Muñoz-Sandoval et al.

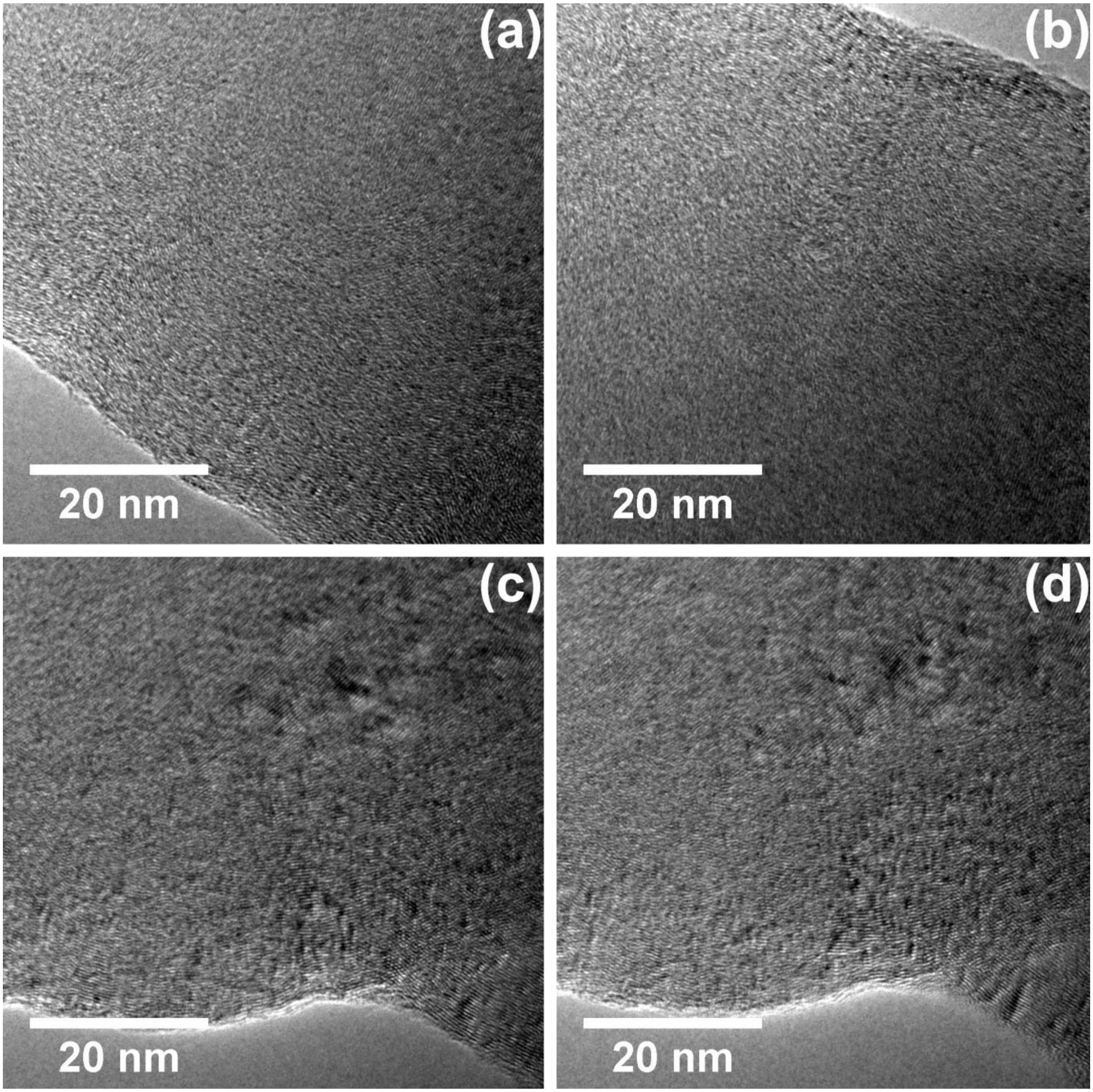

Figure SI-10: HRTEM images showing the outermost layers of the long carbon fibers (LCFs) component of N-CSTNs.
